# Research Progress of Bioactive Components in *Sanghuangporus* spp.

**DOI:** 10.3390/molecules29061195

**Published:** 2024-03-07

**Authors:** Jungu Lu, Manman Su, Xuan Zhou, Deming Li, Xinhui Niu, Yi Wang

**Affiliations:** Department of Regenerative Medicine, School of Pharmaceutical Sciences, Jilin University, 1266 Fujin Road, Changchun 130021, China; lujg22@mails.jlu.edu.cn (J.L.); zhoux21@mails.jlu.edu.cn (X.Z.); lidm23@mails.jlu.edu.cn (D.L.); niuxh22@mails.jlu.edu.cn (X.N.)

**Keywords:** Sanghuang, structure, effects, toxicity, classification, medical properties, polysaccharides

## Abstract

The species in *Sanghuangporus* are a group of edible mushrooms with a long history of oral use in East Asia as a health-improvement method. They should be classified under the genus *Sanghuangporus* rather than mistakenly in *Phellinus* or *Inonotus*. The major components in this genus consist of polysaccharides, polyphenols, triterpenoids, and flavonoids, all of which exist in the fruiting bodies and mycelia. For extraction, studies have shown methods using hot water, ethanol, DES solvent, and alkaline, followed by purification methods including traditional anion column, Sevag solution, macroporous resin, and magnetic polymers. Proven by modern medical technology, these components possess promising anti-inflammatory, antioxidative, antitumor, and immunoregulation effects; additionally, they have health-improving effects including pulmonary protection, hypoglycemic properties, sleep improvement, gout mitigation, antiaging, neuroprotection, and muscle-strengthening abilities. Several toxicity studies have revealed their safety and recommend a dose of 1 g/kg for mice. As a newly emerged concept, functional food can provide not only life-sustaining nutrients but also some health-improving effects. In conclusion, we substantiate Sanghuang as a functional food by comprehensively presenting information on extraction and purification methods, component medical and structural properties, and nontoxicity, hoping to benefit the development of Sanghuang species as a group of functional food.

## 1. Introduction

**Figure 1:**
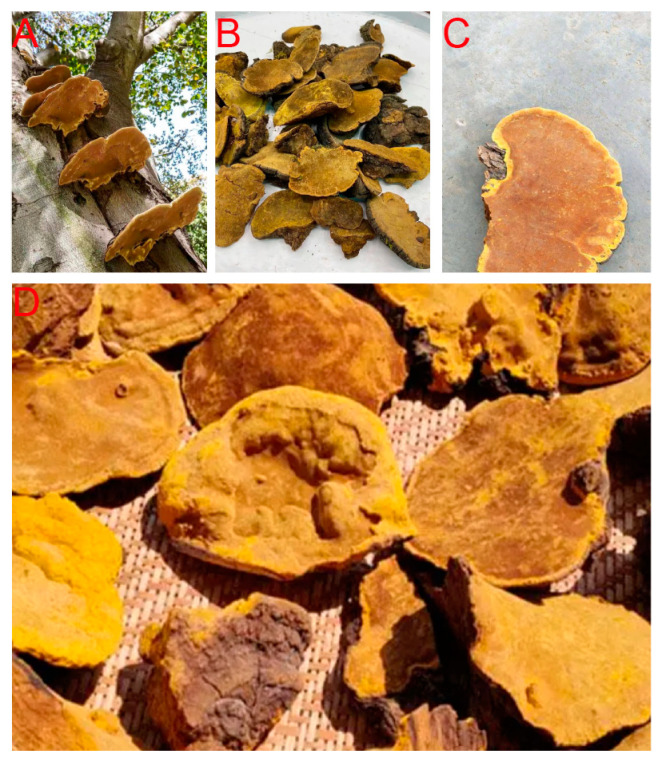
(**A**) The growing state of the Sanghuang species; (**B**–**D**) the dried state of the Sanghuang species.

*Sanghuangporus* spp., also known as *Phellinus* or *Inonotus* in some of the literature and some areas worldwide, is a perennial genus of edible mushrooms widely distributed in boreal forests in East Asia, especially in subtropical and tropical areas, holding profound pharmaceutical potential [1,2,3,4,5,6,7,8]. These medicinal mushrooms, as illustrated in Figure 1, have been popular in oral use since ancient times in countries such as China, Korea, and Japan [4,5,9,10,11,12,13,14,15,16,17,18,19]. The first mention could date back to approximately 2000 years ago, as the name ‘Sanger’ appeared in Shen Nong’s Materia Medica for treating gynecologic tumors taking the forms of abdominal lumps [2,20,21,22,23]. Then, the word ‘Sanghuang’ started to appear in a contemporary medical book called ‘Yao Xing Lun’ in the Tang Dynasty as an oral treatment for what people at the time believed to be cervical cancer, indicated by abnormal vaginal bleeding [14,20,21]. Sanghuang was also mentioned in the Compendium of Materia Medica in the Ming Dynasty as a traditional medicinal mushroom taken orally to eliminate the body’s toxins [2,21]. Entering modern times, pharmaceutical research has demonstrated multiple functions of *Sanghuangporus* spp., including the ability to improve blood circulation, scavenge free radicals, demonstrate anti-inflammatory effects, anti-carcinogenesis, antioxidative abilities, immunomodulatory effects, antidiabetic activities, antimicrobial abilities, and antiaging effects [2,4,9,14,20,21,22,24,25,26,27,28,29]. Some research has also provided evidence of antigout, sleep-improving, neuroprotective, and hepatoprotective activities [3,30,31,32]. Despite their prolonged use in history, research progress on the application of these mushrooms has been slow, mainly due to the following factors. First of all, their classification has been chaotic, with multiple studies presenting their own phylogenetic results. Secondly, few studies have comprehensively gathered key information such as their medicinal properties, extraction, and purification methods. These factors have impeded the scientific use of traditional fungi, preventing its widespread use by more people as a cheap but valuable nutrition source.

The dominant classification for the ‘Sanghuang’ species is as follows: Phylum *Basidiomycetes*, Class *Hymenomycetes*, Order *Polyporales*, Family *Polyporaceae*, and Genus *Sanghuangporus* [25,33]. Over the past few years, some studies have shown that the true *Sanghuangporus* genus contains 15 named species, including the most commonly used three species: *S. baumii*, *S. vaninii*, and *S. sanghuang*. These inhabit Syringa, Morus, and Populus, respectively [25,33]. Despite the fact that not all studies refresh their classifications, this information could still assist future research in providing clearer results with specific species classifications. To comprehensively review information from various sources, this article will include research results that clarify *Sanghuangporus* and those that still regard *Sanghuangporus* as *Phellinus* or *Inonotus*. Throughout this article, the term ‘*Sanghuangporus*’ should be comprehended to refer to formal members of the genus *Sanghuangporus*, and the terms ‘the Sanghuang species’ and ‘Sanghuang’ are used to generally refer to the species mentioned above.

The general process of studying the Sanghuang species often starts from extracting the desired components. To extract them from the Sanghuang species, the fruiting bodies are typically dried and turned into powder, followed by hot water extraction or ethanol extraction, depending on whether the target material comprises water-soluble polysaccharides or other ethanol-soluble components [2,4,34,35,36,37]. These two methods, often assisted by ultrasonic devices, are widely used due to their undeniable advantages: they are cheap and easy to perform [2,13,14,21,32,37,38,39,40,41,42]. But they are also marked by disadvantages, such as long extraction times, low purity or yield, and possible contamination of the environment, which have given rise to some new yet more expensive methods, such as the deep eutectic solvent (DES) method [43]. The mycelia are often acquired through liquid fermentation [15], after which they are used to extract single components, total components, or used as mycelia extracts using the methods mentioned above [3,32,37,44,45,46]. These extracts can be used to study their effects as a whole or further purified using traditional columns, synthetic polymers, or microporous resin, which are discussed in detail in the purification section. Some studies even include structural identification processes for their purified polysaccharides, using methods like infrared (IR) spectrum, methylation analysis, and nuclear magnetic resonance (NMR) spectroscopy [47,48,49]. In the end, the majority of studies focus on the medical properties of these extracts.

Their fruiting bodies and mycelia contain, yet differ in, multiple bioactive components, including polysaccharides, flavonoids, terpenoids, polyphenols, proteins, or saturated fatty acids [5,30,32,47,50]. The fruiting bodies contain mostly water-soluble polysaccharides, whose main monosaccharide composition includes mannose, galactose, glucose, fructose, and xylose [50]. Mycelia, conversely, demonstrate higher levels of polysaccharides, proteins, and other bioactive content than the fruiting bodies [15,50]. The compounds in these two parts provide Sanghuang with many valuable functions, including antitumor activities and free-radical scavenging abilities [6,9,13,16,20,25,26,28,35,37,51,52,53,54]. Polysaccharides are reported to be the major medical component and possess anti-inflammatory, antioxidative, antitumor, diabetes-ameliorating, sleep-improving, and neuroprotective effects, often demonstrated through pathway regulation, free-radical scavenging, and metabolic regulation [3,10,15,32,34,50,55,56,57,58,59]. However, scientists have noticed other components with indispensable medical properties. Hispidin, a polyphenol compound, has been shown by multiple studies to have anticancer and antiviral effects and free-radical scavenging and anti-inflammatory activities [6,21,26,34,35,53,60,61,62,63]. Morin, a flavonoid rarely found in species, possesses significant medical properties like cartilage protection [64,65]. Even some biodegradable fungal pigments could exhibit anticancer, antimicrobial, or antiobesity effects [66].

“Functional foods”, a newly emerged definition in recent years, focuses on a specific kind of food that not only provides life-sustaining nutrition but also improves health or treats diseases [67,68,69,70]. Despite being effective in small doses, most modern medicines have severe side effects, such as organ toxicity or shortening the life span, thus stating the urge to find better supplements or substitutes. Many kinds of mushrooms have now been considered functional foods due to their attractive taste, multiple kinds of beneficial components, and their nutritional value [67,68,69,70]. The secondary metabolites produced by or extracted from them, such as polysaccharides or flavonoids, provide the human body with necessary nutrition and contribute to health improvements, including antioxidant, anticancer, or immunoregulation [71]. Despite the lack of evidence proving Sanghuang is a functional food, its crucial advantages in health improvement and potential for therapeutic advancement have already pointed out the connection [70].

In this review, we summarize the progress in research on the structural and conformational properties of several polysaccharides in hopes of enlightening future research on structure–activity relationships. We conclude and compare different extraction methods and introduce some newly emerging purification methods in detail. Then, we elaborate on the biological activities of Sanghuang components and their possible mechanisms, providing a comprehensive view of how *Sanghuangporus* might be considered a functional food or be helpful in disease treatment.

## 2. Phylogenetic Progress

Species in *Sanghuangporus* have long been classified as *Phellinus linteus*, *Phellinus baumii*, *Phellinus igniarius*, or *Inonotus linteus* by many researchers from China and adjacent countries [6,7,15,16,17,19,30,40,53,60,72,73,74,75,76,77,78,79]. In 2021, Shen et al. presented concrete proof for such misclassification in a study where they revised the ITS sequence in Genbank and clarified some taxonomical mistakes, such as redetermining *I. baumii* and *I. vaninii* as *S. baumii* and *S. vaninii*, respectively [80]. These results provide tremendous aid in future research and enlarge the reference base of *Sanghuangporus* by suggesting that some misclassified species should be *Sanghuangporus*. Misclassification might originate from the following factors: first, a growing interest of non-taxonomists in the medicinal properties of Sanghuang and some errors in taxonomic revisions; second, translating the traditional names in Chinese or Korean into other languages without checking their classification; third, identifying species based only on their morphological characteristics, which can be misleading because of hybridization or convergent evolution, rather than taking genomic analysis into account, which is unique to each species and thus valuable for classification when building phylogenetic trees based on DNA sequencing [80]. This phenomenon confounded some researchers and caused studies on their pharmacological benefits to be rare, thereby hampering further medical applications [30,33]. Therefore, many studies are now focusing on Sanghuang’s taxonomy and building more accurate phylogenetic networks by drawing phylogenetic trees to reveal how closely species are related [33,52,81,82,83]. A phylogenetic tree is built using the standard method, namely phylogenetic systematics, by grouping different species based on shared characteristics, which should suggest uniformity among trees. Yet, the drawing process is subjective due to different understandings of species relationships, thereby creating different phylogenetic trees on the same topic [83].

Over the past few years, researchers have confirmed a reasonable classification of the ‘Sanghuang’ species: Phylum *Basidiomycetes*, Class *Hymenomycetes*, Order *Polyporales*, Family *Polyporaceae*, and Genus *Sanghuangporus,* and that the valid *Sanghuangporus* genus contains 15 named species, including the most commonly used three species, *S. baumii*, *S. vaninii*, and *S. sanghuang*, which inhabit Syringa, Morus, and Populus, respectively [25,33]. To prove that the *Sanghuangporus* exists, Han et al. revised 39 sequences from Genbank and argued that many *Phellinus* species, such as *P. linteus*, were *S. sanghuang* and *S. baumii* [7]. Another group in 2018 conducted a complete mitochondrial genome analysis of *S. sanghuang,* proving that *Phellinus* was not the taxonomically correct name [74,75,76]. These results brought up the importance of clarifying the Sanghuang species. In 2019, Zhu et al. dug deeper and studied their biodiversity, divergence, and phylogeny, after which they expanded the *Sanghuangporus* to four main clades by comparing their morphological similarities, hosts, and living environments [84,85]. Biogeographical estimation showed that the approximate divergence time of the 13 species in their study was in Oligocene in Northeast Asia, possibly caused by global cooling and a gradual replacement of tropical forests by grass [85]. Despite slight variances between results, establishing the Genus *Sanghuangporus* has been widely accepted, suggesting a clearer future in Sanghuang research.

## 3. Extraction Methods

### 3.1. Hot Water Extraction

This traditional method uses hot water as a solvent to boil certain plant parts for hours, usually followed by lyophilization, purification, and adding ethanol or methanol in a few studies for precipitation of crude polysaccharides [1,4,10,11,14,22,45,57,86,87,88,89,90,91,92,93,94]. The phenol sulfuric acid method is then used to measure the content of acquired polysaccharides [90,92,95,96,97,98]. After ethanol precipitation, products mainly contain polysaccharides, whereas the ones lyophilized without precipitation contain other constituents, such as polyphenols [4,24,47,87]. Therefore, ethanol has been most commonly used to assist in water extraction by precipitating polysaccharides from the supernatant [38,55].

Some extracting factors, such as the liquid–solid ratio, the extraction temperature, and the extraction time, can influence the yield of polysaccharides [22]. Yuan et al. stated that the yield of polysaccharides first rose along with the increase in liquid–solid ratio and extraction temperature, and then stopped after extracting for approximately two hours [22]. Researchers use statistical analyses to research on the optimal factor combination, such as designing Box–Behnken tests using the response surface methodology to conduct a cross-analysis between factors [22,39,99,100]. As a well-known for effectively presenting optimized conditions, the response surface methodology can also assist researchers in discovering the extent to which each factor affects the total yield, despite some differences among studies. The results of Yuan et al. suggested that the liquid–solid ratio demonstrated the most decisive influence on the total yield, followed by extraction temperature and extraction time; in contrast, Xu et al., still in consensus with the idea that the liquid–solid ratio has the greatest influence, argued that extraction time demonstrated a more substantial influence than the extraction temperature [22,99].

Assistant methods are used in some studies to make samples more susceptible to extraction or to increase yield. Among them, ultrasonic assistance is most commonly used to process sample powders; during this process, the ultrasound promotes the permeation of solvent molecules into the interstice of tissue cells to ensure that they make full contact with biomolecules [4,36,37,101]. Apart from applying ultrasound, some researchers have applied complex enzymes to assist extraction. Cheng et al. used a composite enzyme including cellulase, pectinase, and protease in a 2:1:1 ratio to extract polysaccharides after soaking samples in ethanol to eliminate triterpenoids [97]. Several other results confirmed that adding composite enzyme could increase polysaccharide yield [98,100]. Some might use microwave combined with ultrasound to assist polysaccharide extraction using hot water [36].

### 3.2. Ethanol Extraction

Ethanol is often utilized when extracting polyphenols, terpenoids, and flavonoids from the Sanghuang species, often assisted by ultrasound [2,13,14,21,32,37,38,39,40,41,42]. Polyphenols and their derivative compounds, such as hispidin, are often best extracted using ethanol as a solvent [3]. However, ethanol extracts also contain other components, such as sesquiterpenoids and carbonyl compounds’ derivatives, suggesting that compounds in the Sanghuang species might not be exclusive to a few categories [32,101]. Some changes of the solvent might occur in some research papers, such as replacing ethanol with 70% methanol or absolute alcohol [9,25,28].

Researchers also use Box–Behnken tests and multifactor level response surface analysis to acquire their optimal conditions in ethanol extractions [37,102]. Cai et al. used ethanol and ultrasound to extract triterpenoids from *S. sanghuang* and ranked factors based on their influence on yield: ethanol concentration, liquid–solid ratio, extraction time, and extraction temperature [37]. They added that increasing extraction time would initially increase the yield yet decrease it at some point, which might result from a prolonged ultrasound permeation that destroyed triterpenoid structures and enhanced the dissolution of other substances [37].

### 3.3. Deep Eutectic Solvent Extraction

Despite the current trend of using ethanol or hot water to extract, these solvents have several disadvantages, such as long extraction time, low purity or yield, and possible contamination to the environment [43]. As a result, a constant search has long been in motion for newer, safer, and more environmentally friendly methods that take less time to finish the extraction while yielding more [43]. DES is a newly emerged extracting method uncovered in 2021 and 2022, which replaces organic solvents like ethanol with a group of environmentally friendly ones acquired by blending two or more solvents or substances in a particular proportion [35,43]. The origin of such a mixed solvent came from the research of Abbott et al., in which they acquired a low-melting eutectic mixture named DES by blending amides and quaternary ammonium salts [43,103]. Compared to old solvents, DES is easy to synthesize, inexpensive, nontoxic, not easy to volatilize, nonflammable, and degradable [35,43].

Similar to the hot water or ethanol extraction methods mentioned above, researchers have utilized some methodologies to ensure the most appropriate conditions. Zheng et al. found an approximate range for each of their experimental factors to yield the best combination by designing single-factor tests [35]. Their highest yield of polyphenols was 1.5 times more than using 60% ethanol, probably due to better viscosity, polarity, and surface tension [35,43]. Zhang et al. added that products extracted with DES showed a better scavenging effect than their ethanol counterparts, pointing out DES’s superiority over ethanol [43]. Therefore, DES methods might draw more attention from researchers in the future.

### 3.4. Alkaline Extraction Methods

Apart from the more commonly used water extraction and ethanol precipitation methods, some researchers have used alkaline to extract polysaccharides from Sanghuang samples [10,45,104]. Evidence has shown that different solvents could influence the final yield, structure, or even bioactivities of polysaccharides [10]. The alkaline solution could destroy the hydrogen bond in cell walls to release the polysaccharides and improve the extraction rate [45]. Building on previous studies, Wang et al. used 1.25 mol/L NaOH/0.05% NaBH_4_ to extract polysaccharides from *P. linteus* mycelia at room temperature for 3 h, followed by neutralization and ethanol precipitation [45]. The polysaccharide content acquired from the phenol–sulfuric acid method was 84.92%. Pei et al. used the same alkaline solution to extract polysaccharides and acquired approximately 90% of polysaccharide content after purification [105]. The higher content compared to the results of Wang et al. might originate from different purification methods. Table 1 concludes the results mentioned above.

**Table 1:** Conclusion and comparison of the extraction methods.

Extraction Method	Material	Product	Purification Method	Optimized Condition			Final Yield	Reference
				Extraction Time	Liquid–Solid Ratio	Temperature/Power		
Ethanol precipitation	Liquid culture broth	4 Exopolysaccharides	DEAE-Sepharose Fast Flow column Sephacryl S-100 HR gel column	-	-	-	11.77% 55.36% 17.12% 15.75%	[48]
Boiling water	Dried fruiting body	Polysaccharide and Polyphenol	-	-	-	-	5.51% 23.00%	[4]
Boiling water	Fruiting body	Polysaccharide	-	2.26 h	21.61:1 mL/g	99.24 °C	9.40%	[22]
Boiling water	Mycelium	Polysaccharide	-	2.5 h	14:1 mL/g	60 °C	4.91%	[99]
Boiling water	Sanghuang powder	Polysaccharide	-	8 h	18:1 mL/g	90 °C	2.12%	[96]
Boiling water	Mycelium	Polysaccharide	-	3.5 h	45:1 mL/g	100 °C	3.99%	[100]
Boiling water	Fruiting body powder	Polysaccharide	DEAE-Sepharose Fast Flow column Sephacryl S-400 column Sephacryl S-200 column	4.35 h	26:1 mL/g	100 °C	-	[106]
Ultrasonication	Mycelium powder	Polysaccharide	-	30 min	40:1 mL/g	45 °C/120 W	10.73%	[39]
		Triterpenoid	-	25 min	50:1 mL/g	45 °C/150 W	1.51%	
Ultrasonication	Mycelium powder	Polysaccharide	-	260 s	49:1 mL/g	464 W	13.19%	[36]
Ultrasonication	Dried fruiting body	Polysaccharide	-	32.7 min	32.5:1 mL/g	360 W	3.46%	[97]
Ethanol extraction	Mycelia and broth of *S. sanghuang*	8 sesquiterpenoids and 6 polyphenols	Sephadex 200 column	-	-	-	-	[101]
Ethanol extraction and ultrasonication	Sanghuang powder	Flavonoid and polyphenol	-	30 min	-	-	(10.18 ± 0.85)% (13.58 ± 1.33)% (14.62 ± 1.05)% (15.38 ± 0.76)%	[21]
Ultrasonication	*S. baumii* powder	Flavonoid and polyphenol	Macroporous membrane	30 min	-	-	-	[28]
Ethanol extraction	Fermented broth of S. lonicericola	Polysaccharopeptide	DEAE exchange column	24 h	-	-	23.00%	[9]
Ethanol extraction	Mycelia of *S. sanghuang*	Triterpenoids	-	20 min	-	60 °C	13.30%	[37]
DES extraction	Dried fruiting bodies of *S. baumii*	Phenolics	-	42 min	34:1 mL/mg	58 °C	12.58%	[35]
DES extraction	Fruiting body	Polyphenols	-	21 min	260:1 mL/g	80 °C	(12.45 ± 1.88)%	[43]

## 4. Purification Methods

### 4.1. Traditional Methods

Multiple studies have demonstrated that chromatography and Sevag reagents are appropriate methods for separating and purifying desired components [9,49,55,101,107]. Sevag reagents have been widely used in purifying fungus polysaccharides as an efficient method to deproteinize or decolorize samples [108,109,110]. For other impurities, such as salts or polysaccharides, researchers have resorted to specific columns based on anion exchange or size exclusion to elute the crude mixture with NaCl or distilled water, which has long been used to purify many compounds from medicinal fungi [5,9,55,90,92,101]. Most studies contained two chromatographic steps using two kinds of columns: diethylaminoethanol (DEAE) chromatography columns (often used first) and Sephacryl columns [5,9,47,48,49,55,92,101,111,112].

### 4.2. Macroporous Resin Methods

The Sanghuang extracts sometimes contain not only proteins or salts but also plant pigments like flavonoids or polyphenols that could not be easily removed with Sevag reagents or columns, thus calling for a more specific and more thorough purification method [86,113,114,115]. With properties like polarity and specific areas, macroporous resin has been developed for the decoloration and purification of crude polysaccharides with a higher efficiency than traditional methods, suggesting the potential for large-scale purification [113,115,116,117]. Evidence has supported the isolation of flavonoids from plant leaves using resins with moderated specific areas [113,115,118,119]. Some studies even pointed out the optimized condition for the resin-purification process. By studying six kinds of macroporous resin on their abilities to absorb Phelligridin LA (PLA) from *I. baumii,* Wang et al. provided optimized conditions: an extraction pH of 5, a proportion of fermentation broth to resin of 4:1, an extraction temperature of 20 °C, an adsorption time of 150 min, and a concentration of PLA of 6.78 mg/mL [118]. Apart from isolating PLA, the microporous resins they used could also assist in quantifying PLA in different samples based on light absorption at 365 nm [118]. The specific ability of microporous resin in polysaccharide decoloration and purification could be significant when applying them to large-scale use and accelerating the pace of Sanghuang commercialization.

### 4.3. Molecular Imprinting Technology

Molecular imprinting technology uses synthesized molecular imprinting polymers (MIPs) to bind to a specific template molecule with high affinity by forming specifically three-dimensional binding sites and certain chemical bonds [64,120,121,122]. Magnetic molecularly imprinted polymers (MMIPs) are a newly emerged version of this technology [64,123,124]. Zhang et al. utilized and improved this method after realizing that traditional isolation methods for morin might be poorly specific and inefficient [64]. In their study, they compared multiple ways to isolate morin, among which the synthetic morin magnetic molecularly imprinted polymers (Morin-MMIPs) showed the highest yield. Apart from a high selectivity, their Morin-MMIPs also showed a short purification time, a good absorption ability, and a high absorption efficiency that remained stable even after six cycles [64]. These polymers could purify relatively rare components more efficiently, thereby facilitating more research on fungus component isolation.

## 5. Conformational Properties of Sanghuang Polysaccharides

**Figure 2:**
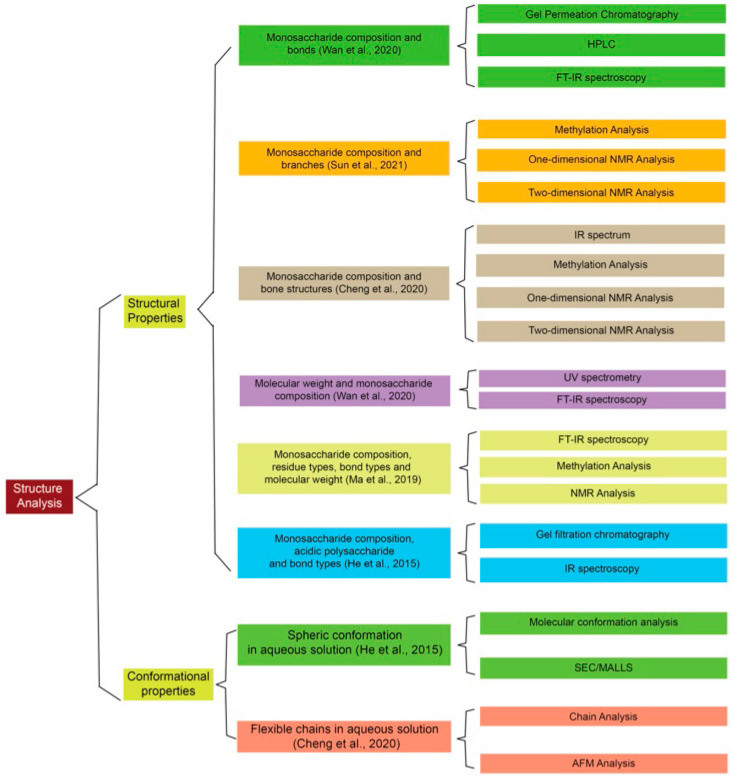
The general methods of identifying the structure of bioactive components from Sanghuang [5,48,49,87,130].

Polysaccharides are common medicinal components in fungi whose composition has led to considerable interest from researchers [55,93,125,126]. The more complex the polysaccharide structure is, the more biological activity is associated with its backbone structure [57,58]. Evidence has already shown that more galactose in polysaccharides leads to more potent anti-inflammatory abilities and that a high concentration of (1,3)-β-glucan in polysaccharides could effectively inhibit intestinal inflammation [87,127]. Other studies have shown that interactions between polysaccharides and proteins might be the critical mechanism of antitumor effects and that polysaccharides might prohibit inflammation progress by inhibiting cytokines [127,128]. The enrichment of branched chains is also reported to reduce inflammatory responses [58]. These promising effects make it paramount to detect and elucidate the structure of Sanghuang polysaccharides, since the Sanghuang species could be considered a functional food. However, no consensus has been established on how Sanghuang polysaccharides contribute to each biological effect. Nor have there been any comprehensive studies on structure–effect relationships. To date, the research related to such relationships has often focused on their case. Therefore, the following section lists several new studies and methods elucidating Sanghuang polysaccharide composition, some digging deeper into structure–effect relationships. Figure 2 includes the main processes and methods required for structure identification.

From 2020 to 2022, Cheng et al. studied the conformational properties and effects of cultured mycelia, fruiting bodies, and liquid culture of *S. sanghuang* [47,48,112]. Building on previous research, they elaborated on the conformational properties of an intracellular polysaccharide named SSIPS1 from the cultured mycelia of *S. sanghuang* and revealed its potential in blood glucose control [47,89]. After monosaccharide composition detection, methylation analysis, and 1D/2D NMR analysis, they inferred that SSIPS1 had a backbone including structures of 1,4-linked α-D-Glcp and two branches separated from the backbone at the O-6 positions that comprised 1,4-linked α-D-Glcp terminated with α-D-Glcp, 1,4-linked α-D-Glcp, and 1,4-linked β-Galp terminated by α-D-Glcp [47]. They inferred that these structures of SSIPS1 might be related to its inhibitory activities against α-amylase and α-glucosidase, and to the ability to increase glucose metabolism in the HepG2 cell model with insulin resistance [47]. Chain conformation analysis and atomic force microscopy (AFM) revealed that SSIPS1 had a flexible chain and a worm-like structure in an aqueous environment; this was also the case for a novel exopolysaccharide named mannan (SSEPS2) from the liquid broth of *S. sanghuang*, mentioned in their studies on its antitumor and cell-proliferation-inhibiting effects [47,48]. They discovered, with the methods listed above, that SSEPS2 had a backbone including 1,3-, 1,2,6-, and 1,2-linked α-D-Manp residues and branches composed of α-D-1,6-linked Manp residues and a terminal α-D-Manp residue [48]. However, this study did not elucidate the relationship between structure and antitumor activities. Then, in 2022, they further extended their research to the fruiting bodies of *S. sanghuang* and acquired a novel mannogalactan (SSPS1) composed of several monosaccharides, such as D-galactose, D-mannose, L-fucose, 3-O-methyl galactose, and D-glucose [112]. Like the two aforementioned polysaccharides, they also discovered the presence of flexible chains in SSPS1 [47,48,112]. But SSPS1 backbone consisted of α (1,6-linked) galactopyronosyl, related to inhibiting tumor cell proliferation [112]. Their study contributed to elaborating on structure–effect relationships and accelerating the process of Sanghuang becoming a functional food. Despite the relatively clear demonstration of these three polysaccharides’ hypoglycemic and antitumor effects, more complex and concrete proof of structure–effect relationships requires future research.

In 2021, Sun et al. isolated a polysaccharide from *P. baumii* fruiting bodies to study relationships between structures and anti-inflammatory effects [87]. Methylation analysis and one-dimensional NMR analysis revealed that the SHPS-1 polysaccharide consisted of 2.2% arabinose, 15.7% mannose, 49.3% glucose, and 32.8% galactose. SHPS-1 also included a backbone containing 1,3-linked β-D-Glcp and 1,6-linked α-D-Galp residues and branched chains separated at the O-6 of the β-D-Glcp residue. They inferred that the backbone structure and residues might contribute to its promising anti-inflammatory effects [87,128]. SHPS-1 also contained more galactose than other polysaccharides isolated from the Sanghuang species, which is possibly related to better anti-inflammatory activities [87,127]. They surmised that the enrichment of mannose on side chains on SHPS-1 might allow it to stimulate macrophage mannose receptors to inhibit inflammatory bowel disease (IBD) [87]. After comparing their results with previous studies, Sun et al. also pointed out that different culturing substrates of *P. baumii* might alter the composition of polysaccharides. Their novel study illustrated the structure of a polysaccharide and gave an assumption of the connection between the anti-inflammatory effects of polysaccharides and their structures, which would enlighten future research.

In 2022, Wan et al. successfully extracted four heterogeneous polysaccharides from *S. vaninii* fruiting bodies, all consisting of 11 different monosaccharides, with glucose and galactose being the main components, comprising 70% of the total [55]. They then focused on one of the polysaccharides, named SVP-1, which consisted of 60.26% glucose, 13.84% mannose, and 11.29% galactose. Methylation revealed the presence of 1,4 and 1,6 connections between SVP-1 glucose groups and between glucose and galactose ones. Fourier transform infrared spectrometry (FTRI) analysis revealed that different eluents might have caused discrepancies between polysaccharides on their monosaccharide composition, molecular weight distribution, functional groups, and uronic acid content among the three acidic polysaccharides, leading to different bioactivities [55]. Wan et al. also confirmed that acidic polysaccharides had promising antitumor effects based on their observation that SVP-1 could inhibit NCI-H460 cell proliferation, corresponding with previous results on other acidic polysaccharides [55,94,129]. However, this result did not show the detailed structure–effect relationship, thus future research is still needed.

Other evidence on Sanghuang polysaccharides has also shown some similar characteristics. Early in 2019, Ma et al. found a polysaccharide free of uronic acid but abundant in D-mannopyranose and glucose [5]. In the same year, Ran et al. isolated an *S. sanghuang* polysaccharide with pyran ring structures, which showed promising antiviral effects and healing-promoting effects after being used in silver nanoparticle synthesis [111]. Instead of forming flexible chains in aqueous environments, a polysaccharide from *S. vaninii*, as He et al. argued, might form a tight spherical conformation with branches in those environments [111,130].

Some studies have already revealed numerous polysaccharide structures and conformation properties from fungi or plants [93,125,126,128]. There is a standard agreement that functional groups or branches are crucial to their medicinal effects, but more detailed relationships between conformational properties and effects still need to be clarified [55]. Researchers have been struggling to identify the core functional groups contributing to the effects from the tremendously complex net of interaction between biomolecules [55,58,131]. Therefore, more evidence could be added to strengthen the ground of the Sanghuang species as functional foods.

## 6. Medical Properties of the Sanghuang Species

### 6.1. Anti-Inflammatory Effects

**Figure 3:**
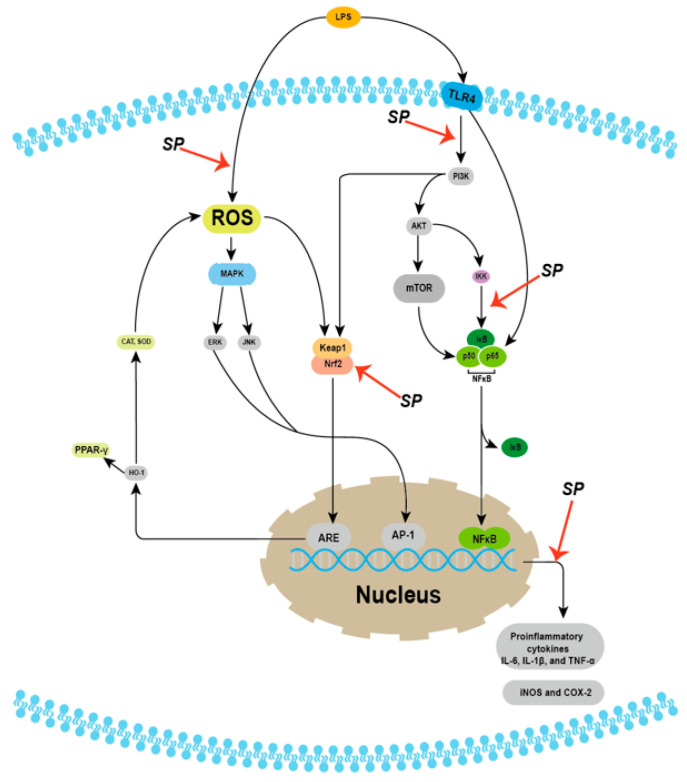
The pathways involved in the anti-inflammatory effects and sleep-improving effects of the Sanghuang species. Red arrows stand for the possible intervention spots of Sanghuang components.

Polysaccharides, flavonoids, polyphenols, and triterpenoids from Sanghuang can affect the inflammation process through several mechanisms [12,18,34,42,87,132,133]. Figure 3 encapsulates several pathways involved in inflammation and where the Sanghuang components might intervene. Many studies have reported an effect on the Phosphatidylinositol-3 kinase/protein kinase B/mammalian target of rapamycin (PI3K/Akt/mTOR) signaling pathway, relating to cell survival [12,13,133,134,135,136,137,138,139]. Akt participates in the signal transduction in lipopolysaccharide (LPS)-induced inflammation by promoting NF-κB activation through IκB kinase (IKK) phosphorylation and regulating downstream activities of mTOR, suggesting that the PI3K/Akt/mTOR pathway could be targeted to reduce proinflammatory cytokine synthesis or NF-κB activation [13,133,135,136,137,140,141,142,143].

Using *S. sanghuang* mycelia ethanol extracts in an ICR mouse inflammation model induced by LPS; both Lin et al. and Jiang et al. mentioned that 500 mg/kg extracts, injected and orally taken, respectively, showed the most robust effects among all doses on reducing the inflammatory status in the lung and liver by activating the PI3K/Akt/mTOR pathway, reducing the permeation of neutrophils, and decreasing the release of proinflammatory cytokines [13,40,132]. Jiang et al. further stated that orally taking 500 mg/kg of the extract for six consecutive days could prevent paracetamol-induced liver damage compared to their positive control group using N-acetylcysteine that could only exert its greatest effect after the damage has been caused, pointing out the clinical potential of Sanghuang [132]. Instead of using extracts, Huang et al. found that 10 mg/kg of purified hispolon, a polyphenol compound from *S. sanghuang*, injected intraperitoneally into the ICR mice one hour before LPS induction, showed the strongest protective effect compared to other doses by decreasing the proinflammatory cytokines, decreasing weight loss, and activating the PI3K/Akt/mTOR pathway [133,144,145,146,147]. The purity of their extracts might explain the dosage discrepancies among these three studies.

Besides corroborating the effects on the TLR4/PI3K/Akt/mTOR pathway, some results have mentioned the activation of other pathways. In 2015, Miao et al. reported that 50 mg/kg *Phellinus* polysaccharides taken orally every day by SD mice with rheumatoid arthritis (RA) might improve the condition of RA by inhibiting the Wnt signaling pathway, based on an observation of a decrease in the expression of β-catenin, C-myc, and ccnd1 [148]. Not many studies have focused on the effects of mushroom components on RA in recent years; therefore, it could be worth researching. In 2017, Lin et al., for the first time, reported that 500 mg/kg of *S. sanghuang* mycelium extract injected intraperitoneally prior to LPS induction could influence the expression levels of Kruppel-associated box (KRAB)-associated protein 1 (KAP1), thereby regulating NF-κB and Nrf2 pathways and further downregulating the syntheses of proinflammatory cytokines [12]. Combined with a previous study in 2008 on the relationship between KAP1- and STAT3-involved pathways, this result introduced the participation of the STAT pathway and the NF-κB and Nrf2 pathways against the inflammation process [12,18,139,149,150,151,152]. Similarly, in 2021, Sun et al. pointed out that treating RAW 264.7 cells with 250 μg/mL of polysaccharide from *P. baumii* for 24 h demonstrated anti-inflammatory effects through the STAT-1 pathway instead of the NF-κB pathway because they failed to observe the expression or translocation of factors in the NF-κB pathway, such as IκBα and p65 [87]. In 2021, Li et al. added that 100 μg/mL hispidin 1 h prior to LPS induction showed the strongest anti-inflammatory effects among all the doses by inhibiting the increase in IL-6, NO production, and TNF-α in cells through nuclear factor erythroid 2-related factor 2 (Nrf2) activation that inhibited inflammation-related gene expression [3].

Despite these results, most studies have observed and reported regulation effects on the TLR4/PI3K/AKT/mTOR signaling pathway and the NF-κB pathway activation, strengthening their crucial role in preventing or inhibiting the inflammation process [12,13,18,132,133,139,153]. NF-κB activation would reduce MAPK-related protein phosphorylation, such as ERK and JNK, and influence the activation of the MAPK to regulate cell migration or proliferation, thereby affecting the levels of downstream proinflammatory cytokines and inflammation-related substances [9,12,13,18,41,86,132,139,154,155,156]. The reduced release of cytokines could prevent or stop causing tissue damage or organ failure, such as edema [13,132,133,157,158,159]. As mostly mentioned by multiple studies, Sanghuang products could effectively reduce IL-6, IL-1β, and TNF-α by decreasing their mRNA levels [3,9,12,13,31,34,41,86,160]. But several studies have also introduced other proinflammatory mediator changes, such as NO decrease and IL-10 mRNA level increase [9,18,95,161]. Taken altogether, the anti-inflammatory abilities and protective effects of an overdose of Sanghuang proven by studies in the past ten years provide strong support for their potential use as a functional food.

### 6.2. Antioxidant Effects

Free radicals and reactive oxygen species (ROS), unstable and detrimental chemicals derived from metabolic pathways in cells with or without specific enzymes, are normally generated through electron transport during respiration or through exposure to exogenous substances, such as tobacco or heavy metals [25,86,162,163,164,165]. Many diseases are related to free radicals and ROS, such as aging, heart diseases, or diabetes mellitus [162,164,165]. The abilities to scavenge free radicals are typically observed and analyzed in vitro by using 2,2-diphenyl-1-picrylhydrazyl (DPPH) and ABTS, creating fade at 517 nm and 734 nm, respectively, with vitamin C (Vc) or butylated hydroxytoluene (BHT) as the positive controls [5,9,25,37,46,96,166]. The color fade can then be observed through spectrophotometry to analyze the radical scavenging abilities of a bioactive component [25,37,46]. Some articles might also add results of scavenging superoxide anions, hydroxyl radicals, or Fe ions, to be more comprehensive [21,37].

Evidence has shown that polysaccharides, polyphenols, flavonoids, and triterpenoids all can scavenge free radicals [21,25,32,35,37,162,167,168,169]. Polyphenols and triterpenoids from the Sanghuang species have long been believed to be major free-radical scavenging components in studies within the 5 years [25,32,35,37,66,162,170,171,172,173]. Cai et al. presented results showing that 18.75 to 350 µg/mL triterpenoid from *S. sanghuang* could increase free-radical clearance from 10% to 90% [37]. Several other studies confirmed that the antioxidant abilities are dose-dependent and better than those of their positive control group [35,162]. However, it remains controversial how strong Sanghuang polysaccharides’ effect is in scavenge free radicals [35,37,73]. In 2019, Li et al. used 4.0 mg/mL *P. igniarius* polysaccharides to scavenge ABTS and to demonstrate the superior antioxidant effects of polysaccharides, whereas others argued that they only played minor roles in antioxidation compared to flavonoids [9,46,73,96,106,174]. A new result in 2022, after comparing 15 strains on their performance of scavenging multiple radicals and reducing power, presented results suggesting that flavonoids and ascorbic acids contribute the most to the antioxidative effect; these are followed by polyphenols and triterpenoids, with polysaccharides being the least-effective antioxidant [173]. However, an article might bring an end to this debate by suggesting a combined antioxidative effect contributed by multiple components instead of a single material [175].

Other components can also demonstrate free-radical scavenging effects. Built on previous research on plant and fungal pigments, in 2018, Heo et al. presented results suggesting that 10 mg/mL extracellular pigments from *S. baumii* possessed higher DPPH and ABTS scavenging abilities compared to other fungal extracts [66,176,177,178,179]. They also gave an original point that some water-soluble polysaccharides from the fruiting bodies or mycelia reported before might be pigments since they had been preprocessed with water several times, so the water-soluble polysaccharides might already be washed away [66,180]. Besides probing into the medical application of extracts, researchers have also been paying attention to fermentation broth or decoction [166,170].

Moreover, some researchers presented results of Sanghuang products affecting antioxidant enzyme activities [5,133,180]. Ma et al. intraperitoneally injected mice with 50, 110, or 170 mg/kg of the exopolysaccharide from *S. sanghuang* broth and 50 mg/kg of Vitamin E for 35 days and argued that the 170 mg/kg dose group demonstrated comparable antioxidative effects to Vitamin E by showing an observable increase in catalase (CAT), superoxide dismutase (SOD), and Trolox equivalent antioxidant (TEAC) content in mouse serum [5]. Instead of using pure extract, Gu et al. discovered that 12 g/kg of *I. sanghuang* decoction taken orally for 15 consecutive days showed an increase in the activities of total antioxidant capacity (T-AOC), SOD, and peroxidase (POD), which was nearly comparable to the Vitamin E control group [181]. They further argued that such antioxidant effects might be related to the Nrf2/HO-1 pathways; this has also been mentioned in other studies before and after them [133,156,181,182]. Built on previous studies, Huang et al. argued that hispolon could increase levels of antioxidant proteins, such as SOD, HO-1, and Nrf-2, and that it elevated the activity of the Keap1/Nrf2/HO-1 and LKB1/CaMKK-AMPK axis to prohibit ROS production induced by LPS [133,137,147].

Researchers have recently agreed that different Sanghuang plants exhibit different antioxidant abilities [22,64,73,86,171,183,184,185,186]. Firstly, evidence suggested that, under the same conditions of being wild or cultivated, *S. vaninii* demonstrated better antioxidant effects and higher total polyphenol and flavonoid content than *S. sanghuang*, despite others arguing to the contrary [21,184,185,187]. Secondly, different cultivation substrates, methods, and years affect antioxidant abilities [23,183,185,187]. A piece of evidence from 2020 showed that adding substances like salicylic acid could increase the content of flavonoids, thereby increasing antioxidant abilities [185]. Thirdly, the antioxidant abilities within the same species could also differ. In 2020, Wang et al. studied several *S. sanghuang* samples and reported that the one tagged SH06 exhibited the most robust DPPH scavenging abilities and reducing power compared to others [185]. Guo et al. added that, even in the same plant, intracellular products showed stronger hydroxyl and DPPH scavenging effects than extracellular counterparts [30]. Finally, different solvents might also affect antioxidant abilities [60,73,86,186]. Despite others arguing to the contrary, in 2016, Yang et al. used *P. igniarius* plants to prove that the water-soluble fractions demonstrated the most potent total antioxidant abilities, with ethanol fractions exhibiting the least potent total antioxidant abilities [188,189].

### 6.3. Antitumor Effects

Evidence has proved that polyphenols and polysaccharides possess cell toxicity effects, antiproliferation effects, and anti-angiogenesis effects on multiple cancer cells in vitro [2,4,15,38,42,72,73,131,190,191,192]. Other components, such as a stable lectin SHL24, could also demonstrate an antitumor effect and remain active when facing many sugars and cations, suggesting a potential for pharmacological applications [193].

There has been a debate on the effectiveness of these components’ antitumor activities, among which flavonoids are found to be more effective than polysaccharides and polyphenols [171,172,185]. Some studies have confirmed that polysaccharides participate in antitumor activities indirectly by stimulating an immune response or directly impeding cell proliferation [4,55,129,194,195,196]. Notably, many studies have provided evidence that Sanghuang polysaccharides could deliver antitumor effects with fewer side effects than traditional medicine, such as cyclophosphamide (CTX), allowing them to be taken as a medicinal foods [14,197,198,199]. CTX worked better in inhibiting tumor growth than Sanghuang polysaccharides while being more toxic, causing immunosuppression [36,200]. Surprisingly, evidence from 2015–2021 has shown that Sanghuang polysaccharides could intensify the effects of CTX and simultaneously mitigate its immunosuppression effects, pointing out not only their superiority as functional food capable of impeding tumor progression but also the potential application of being used with chemical medicine [36,199,200].

Mechanisms for stopping tumor progression have been elaborated further by studies in the last ten years. Impeding or preventing cell proliferation is commonly mentioned in research results, and could be achieved through cell cycle arrest and apoptosis regulation [2,4,20,49,190,197,201,202]. In 2022, Guo et al. stated that 300 ug/mL of their 60% ethanol extracts from *S. vaninii*, basically consisting of polyphenols and flavonoids, demonstrated the most potent anti-proliferation effect by decreasing the cell number in G0/G1 phase by 9.53% and increasing that of G2/M by 9.36% compared to their control group; this effect could be related to inhibiting critical genes in the mTOR signal pathway and relevant protein synthesis [2,72]. Other studies within the 5 years also reported similar changes [20,49,72,86,203,204]. Mechanisms for such arrest might involve regulating expressions of crucial proteins, such as p21, thereby affecting the cyclin-dependent kinase (CDK) complex formation, including the CyclinD-CDK4/6, CyclinE-CDK2, and CyclinA-CDK2 complexes [2,4,72,201,205,206]. On the other hand, studies within the past ten years have proven that Sanghuang components, mostly polysaccharides, could impede cell proliferation by apoptosis, usually involving the Bcl-2 family and the caspase family [4,170,190,201,207,208]. The apoptotic rate of MCF-7 cells after being treated with 0–800 μg/mL S. vaninii polysaccharides rose from 6.1% to 51.8%, in contrast with the rate of living cells dropping from 92.7% to 47.5% compared to the control group; this might be caused by an increased expression of relevant proteins in the caspase family and the mRNA of BAX and p53 [49]. Similar results of downregulated Bcl-2 expression also appeared in several studies in 2021 [4,72,189]. In 2022, He et al. used a dose course of petroleum ether extract of *P. vaninii* containing flavonoids and discovered their 200 mg/kg group had a similar apoptotic effect compared to that of the CTX positive control, which involved forming an equilibrium between BAX and Bcl-2 and the participation of the death receptor pathway [38]. Besides the mechanisms above, Ca^2+^ overload could also cause cells to enter the apoptosis phase by facilitating ER stress, contributing to apoptosis and antitumor effects [20].

Suppressing cell migration could also impede tumor progression and growth [49,107]. Despite not being as good as the positive control Doxorubicin hydrochloride, Wan et al. stated that 800 μg/mL *S. vaninii* polysaccharide could suppress cell migration and leave the scratch area unhealed [49]. Besides polysaccharides, Qiu et al. discovered an antitumor effect of 100 mg/kg S. vaninii Inoscavin A after injecting it intraperitoneally into BALB/c nude mice with HT-29 colon cancer cells, which involved the participation of transmembrane protein smoothened (Smo) receptors and the inhibition of the hedgehog signal pathway [107]. Based on their tumor tissue weight results, they also argue that the antitumor effect of the 100 mg/kg group was weaker than 2 mg/kg of Taxol twice a week [107]. Some studies in the past five years have mentioned that the antimigration effect of Sanghuang is related to the epithelial–mesenchymal transition (EMT) process, causing cells to be less adhesive and more mobile to metastasize to distant regions by inhibiting the matrix metalloproteinase (MMP) from interacting with the extracellular matrix [20,49,72,208,209]. These novel discoveries have shown the possible target of adhesion molecules in cancer treatment.

Other mechanisms also contribute to tumor suppression [49,55]. In 2022, Wan et al. reported that 1000 μg/mL *S. vaninii* polysaccharides inhibited colony formation of non-small-cell lung cancer cells and rendered apparent cell shrinkage and occasional vacuolar cytoplasm compared to other dose groups [55]. Some studies mentioned increasing the intensity of immune responses as a mechanism for inhibiting tumor progression [36,72,198,203,210,211]. Additionally, anti-angiogenesis is related to tumor suppression, which might result from promoting M2 macrophages to polarize to M1 to inhibit vascular endothelial growth factor (VEGF) formation [4,190,191,197,199]. Based on previous results that VEGF binding to VEGF receptors could activate P13K/AKT/mTOR pathways, Zhao et al. inferred that inhibiting VEGF and suppressing essential protein phosphorylation in this pathway might account for the antitumor effects [197].

Disparities exist in antitumor effects among different extracts. Different polysaccharides show different levels of inhibitory effects on tumors [55,212]. In 2017, Ying et al. first reported that mycelium and fruiting body polysaccharides from *P. igniarius*, both 1.5 mg/mL, showed promising proliferation inhibiting effects compared to paclitaxel (10 μg/mL, inhibition rate 72.31%) on HepG2 cells (inhibition rates 66.01% and 70.37%, respectively), suggesting that the fruiting body ones were more effective [212]. Different cultivation conditions also affect the antitumor activities of a given plant. Among the oak segment, sawdust of mulberry branch (BS), and artificial media, the Sanghuang plant grown on BS showed more substantial inhibitory effects on cell reproduction than those grown on others, probably due to a boosting effect of the mulberry branch on *S. vaninii* growth and the accumulation of mycelium components [15,194].

### 6.4. Immunoregulation Effects

Polysaccharides from the Sanghuang species could exert immunoregulation effects by inhibiting the expression of several factors, such as myeloid differentiation factor 88, tumor necrosis factor receptor-associated factor-6, NF-κB, and P38, and by suppressing the production of oxidative radicals and the secretion of cytokines, such as TNF-α and IL-1 [31,56,191,213]. Sanghuang polysaccharides could also increase the level of IL-2 and reduce epidermal growth factor (EGF) expression [170,197,199,211,214].

Apart from affecting factors or cytokines, evidence has also indicated that Sanghuang products could stimulate the proliferation of T and B cells [14,198,199,213,215]. Early in 2003, Kim et al. showed that a 48 h treatment of 200 μg/mL and 500 μg/mL *P. linteus* proteoglycan targeted B cells and demonstrated a promising proliferation effect, though slightly weaker than their positive control of 5 μg/mL LPS [56]. In the next year, Lim et al. discovered that taking 2 mg/day of a *P. linteus* fruiting body extract by oral gavage could regulate T cells by increasing the proportion of splenocytes’ CD^4+^ and CD^8+^ T cells [216]. Instead of using a low concentration, in 2019, Wen et al. used 16 mg/kg of both cultured and wild type *I. sanghuang* chloroform extracts, consisting of polysaccharides and flavones, and orally piped them into mouse stomach for 12 consecutive days and discovered a significantly stronger immuno-regenerative effect than the 4 mg/kg and 8 mg/kg groups by showing more distinctive increase in white blood cells and spleen recovery index and by acting as polyclonal activators of B cells [14]. The higher dosage compared to other studies could be explained by using compound extract or chloroform to extract desired components that could only contain a small amount of polysaccharides, which should be the main component that grants such an immune-regulating effect in Sanghuang. Other evidence showed that the polysaccharides could induce B cells to generate co-stimulatory factors, such as CD80 and CD86 [199,211]. Additionally, polysaccharides could also affect other immune cells, such as peritoneal natural killer (NK) cells, bone marrow cells, and macrophages, thereby increasing the strength of the immune response [86,203]. Taken together, the immunoregulation effect of the Sanghuang species was not limited to the activation of T cells and B cells, which broadened the scope of future research and expanded the possible targets in the commercial application of Sanghuang components.

### 6.5. Antimicrobial Effects

Antimicrobial effects are only sometimes mentioned in most studies. This review concludes a few studies that have mentioned the microbe-suppression effects of polysaccharides, polyphenols, and flavonoids [78,111]. Cheng et al. in 2019 isolated more than a dozen secondary metabolites, mostly sesquiterpenes, from *S. microcystideus* and discovered that 50 μg/mL–100 μg/mL was the minimum inhibitory concentration for most of them, manifold lower compared to each positive control group, suggesting a weak antimicrobial effect against an assay of bacteria, such as *Staphylococcus aureus* and *Escherichia coli* [78]. In the same year, Zhang et al. treated mice infected with *Schistosoma japonicum* by consecutively giving 400 mg/kg ethanol-extracted polysaccharides from *P. igniarius* through oral gavage for 30 days and argued that the fibrosis state and over-expressed TGF-β were ameliorated by both polysaccharide and praziquantel groups through upregulating mRNA expression of *Nrf2* and *Gsta4* gene, suggesting a potential for a long-term functional food source [217]. The polysaccharide from Zhang’s result could work in vivo and bring multiple systems to work, such as triggering the immune system, and cause a combined effect, whereas the extracts in Cheng’s work could only work alone in vitro, which might explain the discrepancy of antimicrobial effects between these two results.

## 7. Health-Improving Effects

Over the years, researchers have focused on utilizing medicines with purified materials or artificial compounds to mitigate or reverse some disease symptoms. However, these medicines might bring some side effects [36,200,218]. Recent studies have shed light on the protective or relieving effects of Sanghuang in several diseases and forms of organ dysfunction.

### 7.1. Pulmonary Protection Effects

Chien et al. has provided up-to-date results in this area [218]. Using a bleomycin-induced mouse model for idiopathic pulmonary fibrosis, they discovered that taking S. Sanghuang orally could significantly decrease fibronectin and collagen expression [218]. Apart from inhibiting fibrosis-related proteins, their results also showed an obvious suppressing effect on inflammation progression, such as inhibiting TNF-α, IL-6, and IL-1β, through regulating key molecules including iNOS, NF-κB, and COX-2 [218]. They further proved the participation of the MAPK pathway by showing a lowering effect on p-ERK, p-JNK, and p-p38 [218]. Moreover, they novelly presented results suggesting that *S. sanghuang* mitigated autophagy and thereby mitigated the progression of idiopathic pulmonary fibrosis [218].

### 7.2. Hypoglycemia- and Diabetes-Mitigating Effects

Diabetes mellitus type 2 (T2DM) is a commonly detected disease connected to organ dysfunctions, insulin resistance, and metabolism disorder, posing a significant threat to public health [34,101,215,219,220,221,222]. During the past few decades, using medicinal foods, such as edible fungi and their bioactive components, to replace traditional medicine in tackling chronic diseases like T2DM has been proven beneficial and has, therefore, received significant attention from researchers [222,223]. Recent discoveries have shown that polysaccharides, triterpenoids, and polyphenols from edible fungi possess antidiabetic and hypoglycemic effects by improving insulin resistance [34,101,224].

Mechanisms of improving insulin resistance have been presented in several studies, including the regulating activities of crucial enzymes, scavenging radicals, to increase abilities to resist antioxidative stress, increasing insulin sensitivities by upregulating the expression of peroxisome proliferators-activated receptor-γ (PPAR-γ), and stimulating B cells to secrete insulin [191,215,225]. Cheng et al. discovered that intracellular polysaccharides from *S. sanghuang* mycelia improved insulin resistance by inhibiting α-glucosidase and α-amylase in vitro and enhancing glucose metabolism in Hep G2 cells [47]. Corroborating the inhibitory effects on these two enzymes, Li et al. discovered that the IC_50_ for ethanol-extracted polysaccharides of α-glucosidase was lower than the positive control, acarbose (0.43 mg/mL and 0.87 mg/mL, respectively), suggesting a better hypoglycemic effect [186]. Yang et al. in 2021 explained the mechanism whereby the blood-sugar-lowering effect might correlate with the IRS1/PI3K/AKT pathway [34].

Apart from improving insulin resistance, products from the Sanghuang species can also affect the concentration of blood lipids, such as triglycerides and total cholesterol steroid [34,60]. Yang et al. stated that 150 mg/kg polyphenols from *P. baumii* could lower HDL-C and LDL-C levels comparably to the 100 mg/kg metformin group [34]. Moreover, in 2019, Huang et al. discovered that consecutively treating mice with streptozotocin (STZ)-induced diabetes with 80 mg/kg polysaccharides from *P. igniarius* for 12 days led to an obvious decrease, similar to their positive control of 4 mg/kg Rosiglitazone, in the fibrosis in kidneys caused by diabetic nephropathy; this could be explained by an upregulated E-cadherin expression, an inhibited α-SMA expression, and a balance between different matrix metalloproteinases [226]. Additionally, a study in 2021 proved that Sanghuang polysaccharides could prevent immune cells from infiltrating into islets, thereby preventing autoimmune diabetes [215].

### 7.3. Sleep-Improving Effects

Traditional ways to improve sleep with medicine have some side effects that might undermine their benefits, such as increasing susceptibility to suicidal ideation [227]. In the year 2021, this situation has changed, since Li et al. brought a new direction for improving sleep [3]. Li et al. used Sprague Dawley rats as an animal model to point out the value of ethanol extracts of *S. sanghuang* mycelia (GKSS) enriched with hispidin as a novel food material for sleep improvement [3]. They gave the rats a regular intake of 70 and 150 mg/kg ethanol extracts of GKSS and acquired their data from electroencephalography (EEG) and electromyography (EMG) electrodes implanted in rats’ brains. The time spent in rapid eye movement (REM) sleep and non-REM (NREM) sleep increased, while the waking time significantly decreased. Based on previous research, they focused on inflammatory cytokines and the Nrf2/ARE signal pathway (Figure 3) [3,153,228]. Besides postponing REM sleep by decreasing IL-6 production, they also observed a dose-dependent activation effect of GKSS and hispidin on ARE-luciferase activities, indicating the activation of the Nrf2/ARE signal pathway and proving the sleep-modulating effects of this pathway [3]. The Nrf2 protein is the connection between the molecular clock and metabolism, thereby influencing circadian gene expression or sleep patterns [3,161,229,230]. These results provide support for commercializing the Sanghuang species as functional food for sleep improvement.

### 7.4. Gout-Mitigating Effects

Gout, a common disease caused by the monosodium urate accumulating in the wrong spots, is usually characterized by high uric acid in the body and related to hyperuricemia [231]. Traditional treatment methods for gout include the used of xanthine oxidase (XOD) inhibitors, such as allopurinol and febuxostat, but they can lead to side effects that could also make patients’ lives difficult, such as kidney failure [231,232]. Therefore, researchers have been trying to find another way to treat gout and have discovered potential in the natural components of plants [232]. In 2021, Guo et al. used 50 mg/kg water extracts of *S. vaninii* to treat mice for nine days and introduced hyperuricemia on day 6 [30]. The aqueous extract demonstrated better effects compared to colchicine in decreasing uric acid levels by downregulating the expression of the catalyst, xanthine oxidoreductase (XOR); this catalyzed the transition of hypoxanthine into xanthine and led to the production of uric acid [30]. They further pointed out that the water extracts might improve the condition of rats with gout arthritis through demonstrating their anti-inflammatory effects, whereby swelling was ameliorated and the level of uric acid in serum was lowered [30].

### 7.5. Antiaging Effects

Aging is related to an imbalance caused by the accumulation of oxidative substances and the degeneration of antioxidative defenses [233]. Metformin is a popular antiaging medicine, but it has side effects [234]. Thus, dietary intervention, such as functional food, is a safe and cost-effective method for people to slow their aging [67,235]. Early in 2019, Wang et al. proved that 200 μg/mL ethanol extracts of *I. obliquus* showed the best antiaging effect in PC12 cell models among other test samples [236]. After using *S. vaninii* methanol and hot water extracts in vitro that mainly contain phenolic and flavonoid components, Li et al. reported an antiwrinkle ability based on the inhibiting effect on tyrosinase, the self-oxidation of L-3,4-dihydroxyphenylalanine (L-DOPA), elastase, and collagenase; these are critical molecules in the body and arterial aging [162,237,238]. Also, in 2019, Gu et al. stated that a Sanghuang decoction consisting of 2 g/mL crude medicine, mostly polysaccharides, triterpenoids, and flavonoids, could initiate the transcription of gene HO-1, thereby inhibiting oxidative stress and delaying the progression of aging [181].

By treating the *Caenorhabditis elegans* nematodes with 10, 25, and 50 mg/mL of *S. sanghuang* extracts to study their effects on lifespan in vivo under the influence of 50 μM 5-fluoro-20-deoxyuridine (FUDR), Dong et al. discovered that, after 14 days, the 25 mg/mL group showed a 26.41% increase in the mean lifespan compared to the control group (20.52 ± 0.34 days and 16.23 ± 0.36 days, respectively) [239]. They also inferred that the antiaging effect was achieved at the mRNA level, such as increased transcriptional regulators of daf-16 and sir-2.1, and that the lifespan was prolonged through the insulin/IGF-1 signaling pathway [239]. Their results, for the first time, revealed the related mechanisms in the antiaging effect of Sanghuang components. This will facilitate the prevalence of the Sanghuang species as a functional food.

### 7.6. Neuroprotective Effects

Neurodegenerative diseases, such as Parkinson’s disease (PD) and Alzheimer’s disease, have become some of the most challenging diseases and have claimed countless lives over the years [68,233,240]. The past decade saw the potential of the Sanghuang species against neurodegenerative diseases, as they could mitigate the damage to nerve cells caused by free radicals [233,240,241,242]. For instance, an ethyl acetate extract of *P. linteus* has been proven to be capable of protecting against neuronal cell death, similar to another result showing polysaccharides from *I. obliquus* protecting HT22 cells in mice with Alzheimer’s disease [243,244]. The Sanghuang species, as a potential functional food, could be of significant assistance in mitigating the development of neurodegenerative diseases.

Parkinson’s disease is marked by a gradual loss of dopaminergic (DA) neurons, causing dysfunction in the neural system and motion abnormalities [15,32,245,246,247,248]. After treating the Zebra fish larvae placed on six six-well plates with 15, 30, and 60 μg/mL of *S. vaninii* mycelia extract, containing compounds such as carboxylic acid derivatives, cinnamic acid and its derivatives, and MPTP as a neuron toxin to induce dopaminergic neuron (DA) malfunction, Li et al. discovered that the 30 μg/mL group had the best performance in ameliorating the loss of DA neurons and vasculature; the 60 μg/mL group showed the most significant improvement in larva locomotor abilities [32]. Based on previous studies on relationships between oxidative stress and PD, Li confirmed that ameliorating oxidative stress assisted the anti-PD effects, such as inhibiting the mRNA expression of several PD-related genes [32,249]. Their results might bring Sanghuang to the frontier of Parkinson’s disease treatment.

### 7.7. Effects on Coronavirus Disease 2019 (COVID-19)

The COVID-19 pandemic is caused by severe acute respiratory syndrome coronavirus 2 (SARS-CoV-2), which is symptomized by fever, cough, and continued muscle pain [42,250,251,252]. As a recent pandemic, it has quickly been elucidated that SARS-CoV-2 uses spike protein to bind angiotensin-converting enzyme 2 (ACE2) to cross the cell membrane, facilitated by the transmembrane protease serine 2 (TMPRSS2) [42,253,254]. It is strategically reasonable to say that viral receptivity could be reduced by blocking protein binding to impede infection [255]. Chien et al. in 2022 discovered that ethanol extracts of *S. sanghuang*, containing several phenolic compounds listed in the article, demonstrated significant and dose-dependent inhibiting effects on ACE2 and TMPRSS2 protein expression in HepG2 cells and 293T cells after 24 h [42]. Their study also showed that the inhibiting effect of 100 mg/kg ethanol extracts in the liver and kidney did not demonstrate liver toxicity, renal toxicity, or pulmonary toxicity; these findings represent the advantages of the Sanghuang species as a potential functional food for ameliorating COVID-19 [42].

### 7.8. Muscle Strengthening Effect

Muscle atrophy is more prevalent among people over 60 years of age than those under 60 years of age due to naturally decreasing muscle mass and strength [256]. The most prevalent treatment for atrophy is neuromuscular electrical stimulation combined with protein [256]. Since members of the Sanghuang species present with promising biological functions, they could be used as daily food to mitigate or prevent muscle atrophy. Early in 2012, Guo et al. reported that mice treated with 400 mg/kg *P. linteus* polysaccharides through intragastrical administration showed significantly increased swimming time within an hour, increasing by 70.62% compared to those given saline [257]. After increasing the dose to 500 mg/kg, they discovered an increase in hepatic glycogen reserve and a reduction in urea nitrogen level in the blood serum, suggesting that the muscle tissue possessed a stronger tolerance to strenuous exercise and a lower susceptibility to atrophy [257]. To corroborate such a fatigue-relieving effect, Li et al. in 2023 orally administrated 200 mg/kg *S. sanghuang* ethanol extract, containing 3 mg/g hispidin, for 39 days to mice with fatigue and discovered a significant improvement in swimming time (178.7%), a 34.36% increase in liver glycogen storage, and an 18% decrease in blood urea nitrogen level [256]. They also presented results that the same concentration could regenerate the mass of muscle with atrophy by a ratio of 89.1%, which was related to MYH4 protein expression [256]. These results indicate that Sanghuang products could be potential functional food with supplement nutrients capable of improving muscle health.

## 8. Toxicity Studies

The effects of bioactive compounds from members of the Sanghuang species make them potential and promising functional foods, only if they are safe for individuals to take in and for cells to absorb. Recent studies have proved their clinical safety [3,24,26,66].

By orally administrating *S. vaninii* aqueous extract to Sprague Dawley rats to perform toxicity studies, Huo et al. reported that the maximum dose for a 21-day acute toxicity study was 21 g/kg and that the 17-week repeated toxicity test indicated 1.0 g/kg to be a safe dose due to no observed adverse effects [24]. They failed to observe any abnormalities in biochemical blood indexes, and organ weights or shapes were detected during the withdrawal and recuperating period, thereby warranting a safe passage for *S. vaninii* use in clinical trials [24]. Another result performed a 28-day oral toxicity study on 80 Sprague Dawley rats by giving them 1, 2, and 5 g/kg of GKSS [3]. The body weight and food intake results did not indicate differences between these three groups [3]. Li et al. also stated that, in addition to there being no deaths or noticeable changes in both sexes, *S. vaninii* hispidin did not cause significant abnormalities in pathology and histopathology. The dosage in these studies was higher than the ones listed in this review, suggesting a higher limit dosage for safe intake of Sanghuang products in mice. Based on some results, the dosage could be pushed to 12 mg/kg [3,26].

Besides in vivo studies to test toxicity on individuals, some researchers have also considered and tested cell toxicity [42]. Li et al. used *S. typhimurium* test strains to study the mutagenic effects of *S. sanghuang* mycelia riching hispidin and found that 3 mg/g mycelia did not induce chromosome aberrations [26]. Their results also showed that the mycelia did not induce abnormal reproduction of reticulocytes, nor did they change the proportion of micronucleated reticulocytes [26]. Back in 2014 and 2017, Zeng et al. and Lin et al. provided similar results that showed no signs of induced chromosome aberrations when testing the toxicity and mutagenic effects of *P. linteus* polysaccharides on bone marrow cells [258,259]. These results indicate the safety of Sanghuang products.

## 9. Potential to Be Functional Food

Mushrooms are prevalent and crucial in humans’ daily lives; for example, they are significantly valuable for cooking [260]. They can provide multiple kinds or forms of minerals, including but not limited to Vitamin B, potassium, and copper [261,262]. Studies in recent years have proved that many edible mushrooms qualify as supplementary or functional foods or possess tremendous medical potential [260,261,262]. Cardwell et al. revealed that mushrooms have the potential to be a convenient source for Vitamin D that could further facilitate calcium absorbance, despite necessary improvements for increasing Vitamin D concentration [262,263]. In order to be a source of nutrition, the species must contain multiple vital or valuable components and be nontoxic, prevalent, and easy to acquire for as many people as possible to use as a supplement. The progress in the past decade, as discussed above, has shown that the Sanghuang species meet the two criteria. They are prevalent and accessible in stores; promising concentrations of polysaccharides, triterpenoids, flavonoids, polyphenols, and sesquiterpenes are the main nutritional components. Additionally, several studies have proven their nontoxic characteristics [3,24,26,66,184]. Given the evidence from recent years listed above, it is logical to assume that the Sanghuang species can be used in many disease treatments or in the amelioration of dysfunction by regulating many pathways. These results propose a new direction for studying the Sanghuang species as a group of functional food.

## 10. Conclusions and Perspectives

Recent studies have proved that members of the Sanghuang species possess tremendous medical potential related to some significant biological components, such as triterpenoids, polysaccharides, flavonoids, and polyphenols. This article concludes the medical properties, including regulation effects on inflammation, oxidation, tumors, and immune responses. Health-improving effects are also concluded, including pulmonary protection, hypoglycemic properties, sleep improvement, gout mitigation, antiaging, neuroprotection, and muscle-strengthening abilities. Multiple in vivo studies have confirmed the safety of consuming Sanghuang components. These results demonstrate their potential to be a group of functional foods.

Multiple studies mentioned extracting components with hot water, ethanol, DES solvent, and alkaline. Hot water is the most prevalent method and it is used to extract water-soluble polysaccharides, whereas researchers use ethanol to extract polyphenols or flavonoids. DES solvent is an environmentally friendly and efficient method that has been developed in recent years. After acquiring crude samples, researchers use traditional chromatography and Sevag reagents to remove proteins or salts or rely on MMIPs and macroporous resins to isolate the desired components from crude samples specifically. Different solvents could present extracts with different antioxidant abilities, probably because different materials could dissolve in different solvents. This article also illustrates disparities in Sanghuang bioactive components because of different species and distribution regions. The extent to which these materials function medically differs, which elicits future research to discriminate and elaborate.

**Figure 4:**
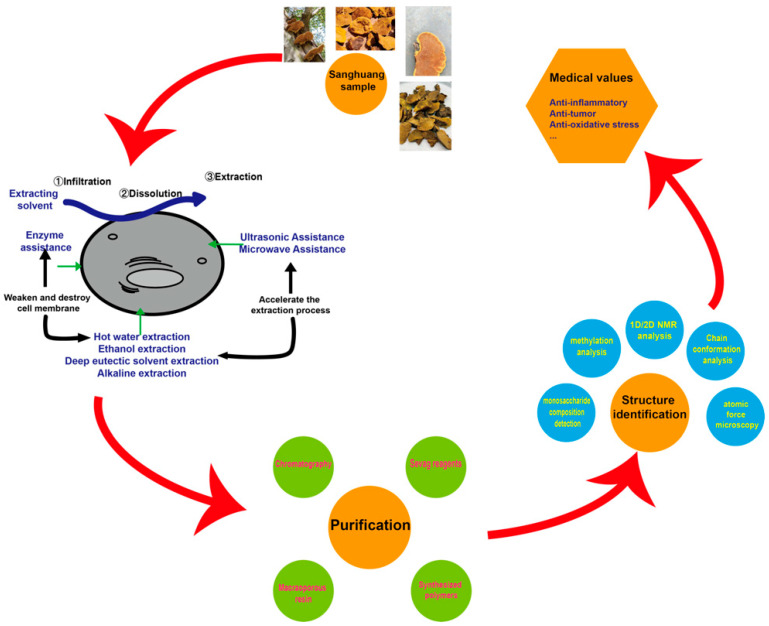
The general process of studying bioactive components from Sanghuang.

As the most prevalent compound, Sanghuang polysaccharides are usually neutral or acidic, including mannose or glucose, and are divided into intracellular and extracellular ones that could form flexible chains in aqueous environments. Studies focusing on structure–effect relationships have found results that suggest that some residues or structures are related to certain biological effects. Flavonoids, triterpenoids, and polyphenols are the primary components in Sanghuang, whose structures have received less attention from researchers and still elude detailed elucidation. These components, as mentioned above, demonstrate the different extents of the effects, such as flavonoids being superior in suppressing tumor growth. They are also produced in different plant parts; therefore, future researchers could isolate certain parts to separate their intended components. Despite recent discoveries that these components could act on several kinds of cells and biomolecules, their exact binding positions and mechanisms of activating pathways still elude understanding. Figure 4 concludes and illustrates the general processes of studying Sanghuang components. Table S1 in the Supplementary Materials presents some results reviewed in this article, including their intended components, extraction methods, the discovered activities, and corresponding signs. These might help future research in studying and consolidating their usage as functional foods.

Numerous studies have focused on the structures, effects, and extraction methods of Sanghuang products. However, little attention has been paid to their phylogeny. Sanghuang has been chaotically and mistakenly classified for years, with species wrongly being assigned to *Inonotus* and *Phellinus*. Such a confounding phenomenon has hampered the dissemination of information or underivative results. Despite a growing number of researchers attaching significance to their phylogenic relationships with each other, the results coming from these studies are too diverse. They comprise several versions of phylogenetic trees without compatibility, which can only provide limited assistance to relevant research. This article listed some research focusing on determining where *Sanghuangporus* fits in evolutionary paths and what species it might include, with methods of genome sequencing and mitochondrial genome sequencing, which might enlighten future research in making further progress in this field.

## Data Availability

No new data were created or analyzed in this study. Data sharing is not applicable to this article.

## Supplementary Materials

The following supporting information can be downloaded at: https://www.mdpi.com/article/10.3390/molecules29061195/s1.

## Abbreviations

DPPH	2,2-diphenyl-1-picrylhydrazyl
ABTS	2,2′-azino-bis
IR	infrared
AFM	atomic force microscopy
ITS	internal transcribed spacer
IBD	inflammatory bowel disease
FTRI	Fourier transform infrared spectrometry
DES	Deep eutectic solvent
DEAE	diethylaminoethanol
PLA	Phelligridin LA
MIPs	molecular imprinting polymers
MMIPs	magnetic molecularly imprinted polymers
VTES	Vinyltriethoxysilane
Fe_3_O_4_ NPs	Fe_3_O_4_ nanoparticles
ROS	oxygen species
CAT	catalase
SOD	superoxide dismutase
TEAC	Trolox equivalent antioxidant
T-AOC	total antioxidant capacity
POD	peroxidase
LPS	lipopolysaccharide
CTX	cyclophosphamide
IKK	IκB kinase
KAP1	Kruppel-associated box (KRAB)-associated protein 1
RA	Rheumatoid Arthritis
Vc	vitamin C
BHT	butylated hydroxytoluene
CDK	cyclin-dependent kinase
