# Supplementary material for: Research Progress of Bioactive Components in Sanghuangporus spp
Source: Molecules. 2024 Mar 7;29(6):1195. doi: 10.3390/molecules29061195 (PMC10976032; doi:10.3390/molecules29061195)
Supplement: Supplementary file 1 [file molecules-29-01195-s001.zip › molecules-2848980-supplementary.pdf]

Table S1

Bioactive components or parts	Abbreviation	Source	Acquiring methods of bioactive components or parts	Activity	Experimental materials (including animals or cell line)	Experimental method	Mechanism or sign(s)	Relevant pathway(s) or protein(s)	Ref.
Mycelium extract	SS-1	Mycelium (*S. sanghuang*)	Extracted with 70% ethanol	Anti-inflammatory	RAW264.7 cells Male ICR mice	in vitro and in vivo	TNF-α(-), IL-1β(-), IL-6(-), NO(-), IL-10(+)	MAPK TLR4/PI3K/Akt/ mTOR/IKKβ	[12]
Mycelium extract	SS	Mycelium (*S. sanghuang*)	Extracted with 70% ethanol	Anti-inflammatory	Male ICR mice	in vivo	NO(-), TNF-α(-), IL-1β(-), IL-6(-) iNOS(-), COX-2(-)	TLR4/PI3K/Akt axis pathway CaMKKβ/LKB1/ AMPK pathway	[132]
Phenolic compounds	PPE	Fruiting bodies (*P. baumii*)	Extracted with ethyl acetate	Anti-inflammatory	RAW264.7 cells	in vitro	TNF-α(-), IL-1β(-), IL-6(-)	—	[34]
Hispolon	—	—	—	Anti-inflammatory	Male ICR mice	in vivo	TNF-α(-), IL-1β(-), IL-6(-), NO(-), iNOS(-), COX-2(-)	IKK/IκBα/NF-κB signaling pathway MAPK pathway TLR4/PI3K/Akt/ mTOR Axis	[133]
Polysaccharides	SHPS-1	Fruiting bodies (*P. baumii*)	Extracted with hot water at 100°C	Anti-inflammatory	RAW264.7 cells Male C57BL/6 mice	in vitro and in vivo	NO(-), iNOS(-), TNF-α(-), IL-1β(-), IFN-γ(-), IFNβ(-), IL-10(+),	STAT-1 pathway	[87]
Extracellular polysaccharopeptide	SePSP	Mycelium (*S. lonicericola*)	Precipitated with absolute ethanol	Anti-inflammatory	Male C57BL/6J mice	in vivo	TNF-α mRNA(-), IL-1β mRNA(-), IL-10 mRNA(+)	—	[9]
Mycelia	GKSS	Mycelia (*S. sanghuang*)	Extracted with ethanol	Anti-inflammatory	RAW 264.7 macrophages	in vitro	NO(-), TNF-α(-), IL-1β(-), IL-6(-)	Nrf2 pathway	[3]
Polysaccharides	—	*S. vaninii*	Extracted with hot water and ultrasonic divices	Anti-inflammatory	Human normal liver cell line L02	in vitro	Myeloid differentiation factor 88(-), NF-κB(-), TNF-α(-), IL-1α(-), IL-1β(-), IL-6(-)	—	[30]
Water extracts and ethanol extracts	—	*P. igniarius* *P. linteus*	Extracted with hot water at 80°C and 95% ethanol	Anti-inflammatory	BV2 cells	in vitro	NO(-)	—	[95]
Polyphenols	ISE	*I. sanghuang*	Extracted with ethyl acetate	Anti-inflammatory	RAW264.7 macrophages	in vitro	TNF-α(-), IL-6(-), NO(-)	MAPK pathway STAT-3 pathway	[18]

Polysaccharides	—	Mycelium (*P. igniarius*)	—	Anti-inflammatory	Male Wister rats	in vivo	TNF-α(-), IL-1β(-), IL-17(-) IL-10(+)	NF-κB pathway	[154]
Ethanol extracts	SS	Dried basidiocarp powders (*S. sanghuang*)	Extracted with 70% ethanol	Anti-inflammatory	RAW264.7 Male ICR mice	in vitro and in vivo	TNF-α(-), IL-1β(-), IL-6(-), NO(-), iNOS(-), COX-2(-), TLR4 and PI3K protein expression(-)	TLR4/PI3K/Akt/mTOR/IKKβ signaling pathway	[13]
Polysaccharides	PPS	—	Extracted with hot water at 80°C	Anti-RA	Male Sprague Dawley rats	in vivo	SFRP1(+), SFRP2(+), fibronectin (-), β-catenin(-), C-myc(-), ccnd1(-)	Wnt signaling pathway	[148]
Water extracts and methanol extracts	HE and ME	Fruiting bodies (*P. vaninii*)	Extracted with hot water and 80% methanol	Anti-oxidant	Free radicals	in vitro	Lipid peroxidation(-), DPPH(-), hydroxyl radical(-), xanthine oxidase(-)	—	[162]
Mycelium extract	SvMEs	Mycelium (*S. vaninii*)	Extracted with 50% ethanol by 35 kHz ultrasonic assistance	Anti-oxidant	Free radicals, Zebrafish	in vitro and vivo	DPPH(-), hydroxyl radical(-), fluorescence signals(+)	—	[32]
Total triterpenoids	—	Mycelium (*S. sanghuang*)	Extracted with ethanol by ultrasonic assistance	Anti-oxidant	Free radicals	in vitro	Superoxide anions(-), ABTS(-), DPPH(-), hydroxyl radical(-)	—	[37]
Phenolics	—	Fruiting bodies (*S. baumii*)	extracted with DES solvent	Anti-oxidant	Free radicals	in vitro	ABTS(-), DPPH(-), hydroxyl radical(-)	—	[35]
Exopolysaccharide	SHP-2	Fermentation broth (*S. sanghuang*)	Concentration	Anti-oxidant	Male or female Kun Ming mice	in vivo	CAT activity(+), SOD activity(+), TEAC activity(+), lipofuscin(-)	—	[5]
Polysaccharides	—	*P. linteus*	Extracted with hot water at 80°C	Anti-oxidant	Free radicals	in vitro	DPPH(-), hydroxyl radical(-)	—	[96]
Polysaccharides	PP	Mycelium (*P. igniarius*)	Centrifugation	Anti-oxidant	Free radicals	in vitro	ABTS(-), DPPH(-)	—	[46]
Polysaccharides	—	Fruiting bodies (*P. baumii*)	Extracted with ethanol by 53 kHz ultrasoni	Anti-oxidant	Free radicals	in vitro	DPPH(-), hydroxyl radical(-)	—	[106]

			c assistance						
Pigments	—	*S. baumii*	Extracted with 95% ethanol	Anti-oxidant	Free radicals	in vitro	ABTS(-), DPPH(-)	—	[66]
Polyphenols and triterpenoids	—	Mycelia (*S. vaninii*)	Extracted with methanol and acetone	Anti-oxidant	Free radicals	in vitro	DPPH(-)	—	[44]
Flavonoids in fermentation broth	—	Fermentation broth (*I. sanghuang*)	Filtration	Anti-oxidant	Free radicals	in vitro	ABTS(-), DPPH(-), hydroxyl radical(-)	—	[166]
Total flavone contents	—	*Phellinus sp.*	Extracted with methanol by ultrasonic assistance	Anti-oxidant	Free radicals	in vitro	Superoxide anion(-), hydroxyl radical(-)	—	[172]
Fermentation broth	—	*S. vaninii* *S. baumii* *I. hispidus*	Centrifugation	Anti-oxidant	Free radicals	in vitro	DPPH(-), hydroxyl radical(-)	—	[183]
Flavonoids and Polyphenols	—	*S. vaninii* *S. baumii* *S. sanghuang*	—	Anti-oxidant	Free-radical scavenging Ferric ions	in vitro	ABTS(-), Ferric ion(-)	—	[184]
Flavonoids and Polyphenols	—	*S. sanghuang* *S. vaninii*	Extracted with 70% ethanol by ultrasonic assistance	Anti-oxidant	Free-radical scavenging β-caratene bleaching experiment	in vitro	DPPH(-), hydroxyl radical(-), absorbance at 470nm(-)	—	[21]
Fermentation products	—	*S. sanghuang* *S. vaninii*	Extracted with 70% ethanol by ultrasonic assistance	Anti-oxidant	Free-radical scavenging	in vitro	DPPH(-), hydroxyl radical(-)	—	[187]
Flavonoids in fermentation broth	SH	*S. sanghuang*	Liquid fermentation	Anti-oxidant	Free-radical scavenging total reducing power analysis	in vitro	DPPH(-), hydroxyl radical(-) absorbance at 700nm(+)	—	[185]

Extracellular products and intracellular products	ExPs and InPs	*S. vaninii*	Extracted with hot water by ultrasonic assistance	Anti-oxidant	Free-radical scavenging total reducing power analysis Human normal liver cell line L02	in vitro	Ferric irons(-), Ferrous irons(+), DPPH(-) Xanthine oxidoreductase(-), Hypoxanthine-guanine phosphoribosyltransferase(+)	—	[30]
Water extracts and ethanol extracts	—	*P. igniarius*	Extracted by decoction and with alchohol	Anti-oxidant	Free-radical scavenging Ferric ions	in vitro	Ferric iron(-), DPPH(-)	—	[98]
Petroleum ether fractions ethyl acetate fractions ethanol fractions water soluble fractions	—	*P. igniarius*	Extracted with water, ethanol, ethyl acetate, and petroleum ether	Anti-oxidant	Free-radical scavenging Ferric ions	in vitro	Ferric iron(-), DPPH(-), hydroxyl radical(-)	—	[188]
Crude polysaccharides and ethanol extracts	—	*S. vaninii*	Extracted with ethanol by ultrasonic assistance	Anti-oxidant	Free-radical scavenging	in vitro	ABTS(-), DPPH(-), hydroxyl radical(-)	—	[186]
Polysaccharides	PIP-1	*P. igniarius*	Extracted with deionized water	Anti-oxidant	Free radicals Ferrous metal ion chelating activity	in vitro	DPPH(-), hydroxyl radical(-)	—	[90]
Polysaccharides	PL-N	Mycelia (*P. linteus*)	Extracted with alkaline	Anti-oxidant	Free-radical scavenging Male ICR mice	in vitro and in vivo	DPPH(-), hydroxyl radical(-), organ indices(+), superoxide dismutase(+), catalase(+), glutathione peroxidase(+), malondialdehyde(-)	—	[45]
Polysaccharides	—	Fruiting bodies (*P. igniarius*)	Extracted with hot water	Anti-oxidant	Free-radical scavenging Iron ion chelaing capacity analysis	in vitro	DPPH(-), absorbance at 562nm(-)	—	[22]

Polyphenols	—	Fruiting bodies (*S. vaninii*)	extracted with DES solvent	Anti-oxidant	Free-radical scavenging	in vitro	ABTS(-), DPPH(-)	—	[43]
Polysaccharides	PLP1-I	Mycelia (*P. linteus*)	Extracted with hot water	Anti-oxidant	Female ICR mice	in vivo	SOD(+), GSH-Px(+), CAT(+), MDA(-)	—	[88]
Polysaccharides	—	*P. linteus*	Extracted with hot water	Anti-oxidant	Free radicals	in vitro	DPPH(-), lipid peroxidation(-), ferric iron(-)	—	[10]
Hispidin	—	Liquid culture broth of *P. linteus*	Extracted with ethyl acetate	Anti-oxidant	Free radicals	in vitro	DPPH(-), ABTS(-)	—	[62]
Polysaccharides	PBMP	Mycelia (*P. baumii*)	Extracted with distilled water	Anti-oxidant	Female Kunming mice	in vivo	SOD(+), CAT(+), GSH-Px(+), TAOC(+), MDA(-)	—	[180]
Ethanol extracts	SS	Dried basidiocarp powders of *S. sanghuang*	Extracted with 70% ethanol	Anti-oxidant	Male ICR mice	in vivo	GPX(+), SOD(+), CAT(+), Trx-1(+), cytoplasm of Nrf2(+)	MAPK pathway	[13]
Polyphenols	SH	Mycelia and broth (*S. sanghuang*)	Extracted with ethanol	Anti-oxidant	Free-radical scavenging	in vitro	ABTS(-), DPPH(-)	—	[101]
Decoction	—	*I. sanghuang*	Extracted by decoction	Anti-oxidant Anti-aging	ICR mice (aging model)	in vivo	Serum MDA(-), serum T-AOC(-), serum GSH(-) Liver POD(+), liver T-AOC(+), liver SOD(+)	Nrf2/HO-1 pathway	[181]
Ethanol extracts	—	*P. baumii*	Extracted with 95% ethanol	Anti-oxidant Anti-aging	PC12 cells MTT assay	in vitro	Superoxide anion(-), hydroxyl radical(-) absorbance at 490nm(+)	—	[236]
Water extracts and methanol extracts	HE and ME	Fruiting bodies (*P. vaninii*)	Extracted with hot water and 80% methanol	Anti-aging	Chemical reaction B16-F10 cells	in vitro	Tyrosinase(-), L-DOPA auto-oxidation(-), melanin synthesis(-), elastase(-), collagenase(-)	—	[162]
Polysaccharides	—	Fruiting body and mycelium (*P. igniarius*)	Extracted with hot water by ultrasonic assistance	Anti-tumor	HepG2 cells S-180 sarcoma strain	in vitro and vivo	Proliferation rates(-) Tumor growth(-)	—	[212]
Extracts	—	Fruiting bodies (*P. vaninii*)	Extracted by decoction and with water and	Anti-tumor	ICR mice	in vivo	Tumor weight(-), tumor cell loosely arranged(+), vacuoles(+), Bcl-2(+), BAX(+), TNF-α(+), IFN-γ(+),	—	[38]

			petroleum ether				caspase-3(+), caspase-9(+), NF-κB(+), JAK(+), P-Akt(-)		
Polysaccharides	—	Mycelium (*P. igniarius*)	Extracted with water	Anti-tumor	KM mice	in vivo	Body weight(+), tumor cell swollen(+), IL-2(+), IL-6(+), TNF-α(+)	—	[198]
Polysaccharides	—	Mycelium (*P. igniarius*)	Extracted with water	Anti-tumor	Male KM mice	in vivo	Body weight(+), tumor cell swollen(+), IL-6(-), TNF-α(-),VEGF(-), IL-1β(+)	PI3K/AKT/mTOR signaling pathway	[197]
Intracellular polysaccharides	IPS	Mycelia (*P. igniarius*)	Extracted with hot water	Anti-tumor	HepG2, SW480 cells	in vitro	Cell growth(-),	—	[92]
Polysaccharides	PL-N1	Mycelia (*P. linteus*)	Extracted with alkaline	Anti-tumor	HepG2 cells	in vitro	Cell growth(-),	—	[105]
Polysaccharides	PIP-1	*P. igniarius*	Extracted with deionized water	Anti-tumor	HepG2, SW480 cells	in vitro	Cell growth(-),	—	[90]
Aqueous extracts	SH	Fruiting bodies (*S. vaninii*)	Extracted with boiling water	Inhibiting cell proliferation	A375 melanoma cells	in vitro	Cell proliferation(-), S phase arrest, p21(+), p27(-), CyclinA(-), CyclinC(-), CyclinD(-), CyclinE(-), CDK1(-), CDK2(-), CDK4(-), CDK5(-), CDK6(-), E2F1(-), DP1(-)	p21-cyclin-CDK complex signalling pathway	[4]
Polysaccharides	PP	*P. linteus*	—	Inhibiting cell proliferation	U-251MG cell line	in vitro	Cyclin A(-), CDK4(-)	—	[190]
Ethanol extracts(mostly polyphenols and flavonoids)	—	*S. baumii* grown on 3 substrates	Extracted with 95% ethanol	Inhibiting cell proliferation	NCI-H460 cells PC3 cells MDA-MB-231 cells SMMC-7721 cells BEL-7402 cells SH-SY5Y cells	in vitro	Proliferation rates(-)	—	[194]
Polysaccharides	SVPs	Fruiting bodies (*S. vaninii*)	Extracted with hot water	Inhibiting cell proliferation	Three NSCLC cell lines (95-D, A549 and NCI-H460)	in vitro	Proliferation rates(-)	—	[55]
Water, 60% ethanol, and	SVW, SVE60, SVE95	*S. vaninii*	Extracted with water,	Inhibiting cell	SW480 cell line	in vitro	Cell proliferation(-), cell survival rate(-), Cells in S	mTOR signalling pathway	[2]

95% ethanol extracts			60% ethanol and 95% ethanol	proliferation			phase(+), cells in G0/G1 phase(-), cells in G2/M phase(-)		
Polysaccharides	SVP	Fruiting bodies (*S. vaninii*)	Extracted with boiled water	Inhibiting cell proliferation	MCF-7 cells	in vitro	Proliferation rates(-), p21(+), Cell in G2/M phase(-)	—	[49]
Extracts	ESV	Fruiting bodies (*S. vaninii*)	Extracted with 95% ethanol	Inhibiting cell proliferation	human cervical cancer cells (SiHa)	in vitro	Cell in G0/G1 phase(+)	—	[20]
Flavonoids	—	Mycelium (*P. igniarius*)	Extracted with methanol	Inhibiting cell proliferation	HepG2 cells	in vitro	Cells in G1 phase(-), Cells in S and G2 phase(+)	—	[204]
Inoscavin A	—	Sporocarps (*S. vaninii*)	Extracted with 95% ethanol by ultrasonic assistance	Inhibiting cell proliferation	Male BALB/c nude mice HT-29 cells	in vitro and vivo	Tumor size(-), tumor volumes(-), tumor weights(-), Smo and Gli1 expression(-), Shh(-), Ptch1(-), Smo and Gli1(-), Smo(-), Smo receptor(-)	Hedgehog signaling pathway	[107]
Polysaccharides	SVP-1	Fruiting bodies (*S. vaninii*)	Extracted with hot water	Altering cell morphology and colony formation	NSCLC cells	in vitro	Cell shrinkage(+), occasional vacuolar cytoplasm(+)	—	[55]
Polysaccharides	SVP	Fruiting bodies (*S. vaninii*)	Extracted with boiled water	Inhibiting cell migration	MCF-7 cells CCK-8 assay	in vitro	MMP-2 mRNA(-), MMP-9 mRNA(-)	—	[49]
Polysaccharides	PP	*P. igniarius*	—	Inhibiting cell migration	U-251MG cell line	in vitro	Absorbance at 490nm(-), migration rate(-), perforation rate(-), PI3K(-), Akt(-), p-PI3K(-), p-Akt(-)	PI3K/Akt signaling pathway	[190]
Mannogalactan	SSPS1	Fruting bodies (*S. sanghuang*)	Extracted with distilled water	Apoptosis	HepG2 cells	in vitro	Apoptic rates(+), Bax expression(+), Bcl-2 expression(-)	—	[112]
Inoscavin A	—	Sporocarps (*S. vaninii*)	Extracted with 95% ethanol by ultrasonic assistance	Apoptosis	HT-29 cells	in vitro	Smo(-), Smo receptor(-)	Hedgehog signaling pathway	[107]
Polysaccharides	SVP-1	Fruiting bodies (*S. vaninii*)	Extracted with hot water	Apoptosis	NSCLC cells	in vitro	Blue fluorescence signal(+), apoptotic bodies(+)	—	[55]

Aqueous extracts	SH	Fruiting bodies (*S. vaninii*)	Extracted with boiling water	Apoptosis	A375 melanoma cells	in vitro	Pro-apoptotic genes (Bad, Bik, Bid, Bim and Bak)(-), Bcl-2(-)	—	[4]
Water, 60% ethanol, and 95% ethanol extracts	SVW, SVE60, SVE95	*S. vaninii*	Extracted with water, 60% ethanol and 95% ethanol	Apoptosis	SW480 cell line	in vitro	Late apoptic cells(+), early apoptic cells(+)	mTOR signalling pathway	[2]
Extracts	ESV	Fruiting bodies (*S. vaninii*)	Extracted with 95% ethanol	Apoptosis	human cervical cancer cells (SiHa)	in vitro	Apoptic rates(+), ER stress(+), intracellular Ca2+(+)	—	[20]
Polysaccharides	SVP	Fruiting bodies (*S. vaninii*)	Extracted with boiled water	Apoptosis	MCF-7 cells CCK-8 assay	in vitro	Apoptic rates(-), Bax mRNA(+), p53 mRNA(+), Bcl-2(-), caspase-3(+), cleaved caspase-3(+), caspase-9(+), cleaved caspase-9(+), caspase-8(+), Bax(+), p53(+)	Bcl-2/Bax pathway	[49]
Polysaccharides	PP	*P. linteus*	—	Apoptosis	U-251MG cell line	in vitro	Cyt-c(+), Caspase3(+)	—	[190]
Ethanol extracts(phelonic compounds)	SS	Mycelia (*S. sanghuang*)	Extracted with 70% ethanol	Anti-COVID-19	HepG2 and 293T cell lines C57BL/6 female mice	in vitro and in vivo	ACE2 expression(-), TMPRSS2 expression(-)	—	[42]
Mycelia	GKSS	Mycelia (*S. sanghuang*)	Extracted with ethanol	Sleep improving	Male Sprague Dawley rats	in vivo	NREM sleep(+), REM sleep(+)	Nrf2 pathway	[3]
Hispidin	—	Liquid culture broth (*P. linteus*)	Extracted with ethyl acetate	Anti-diabetic	RINm5F cells	in vitro	Hydrogen peroxide inhibited insulin secretion	—	[62]
Polyphenols	SH	Mycelia and broth (*S. sanghuang*)	Extracted with ethanol	Increasing glucose upake	HepG2 cells	in vitro	2-NBDG uptake(+), glucose uptake(+)	—	[101]
Polysaccharides	—	*P. igniarius*	Extracted with hot water at 80°C	Improving conditions of type 1 diabetes	KM mice	in vivo	Oxidative stress(-), lipid metabolism(+), food intake(+), gluconeogenesis(-), body weight(+), glucose-tolerance(-), organ indices(+)	—	[225]
Intracellular polysaccharides	SSIPS1	Mycelia (*S. sanghuang*)	Extracted with hot water at 90°C	Hypoglycemic	HepG2 cells	in vitro	α-glucosidase(-), α-amylase(-), glucose consumption(+), hexokinase(+), pyruvate kinase(+)	—	[47]

Crude polysaccharides and ethanol extracts(mostly polyphenols)	—	*S. vaninii*	Extracted with ethanol by ultrasonic assistance	Hypoglycemic	Chemical reaction in the solution containing α-glucosidase and α-amylase	in vitro	Absorbance at 540nm(-), Absorbance at 405nm(-)	—	[186]
Phenolic compounds	PPE	Fruiting bodies (*P. baumii*)	Extracted with ethyl acetate	Hypoglycaemic	Male ICR mice	in vivo	Weight gain(-), blood glucose(-), glucose-tolerance(-), insulin resistance index(-), glucagon(-), epinephrine(-), glycosylated hemoglobin(-), TG(-), HDL-C(-), LDL-C(-)	IRS1/PI3K/AKT signalling pathway	[34]
Crude polysaccharides and ethanol extracts	—	*S. vaninii*	Extracted with ethanol by ultrasonic assistance	Hypouricemic	Chemical reaction in the solution containing xanthine oxidase	in vitro	Xanthine oxidase(-)	—	[186]
Extracellular products and intracellular products	ExPs and InPs	*S. vaninii*	Extracted with hot water and ultrasonic divices	Hypouricemic and anti-gout	Human normal liver cell line L02 Male Wistar rats Female BALB/c mice	in vitro and vivo	XOR(-), HGPRT(+) Swelling(-), serum UA(-)	—	[30]
Polysaccharides	PIP	*P. igniarius*	Extracted with hot water at 90°C	Improving conditions of diabetic nephropathy	Male C57BL/6 mice	in vivo	Urinary protein 24h(-), blood urea nitrogen(-), serum creatinine(-), fasting blood sugar(-), collagen deposition(-), E-cadherin(+), α-smooth muscle actin(-), MMP-2(+), tissue inhibitor of metalloproteinase-2(-)	P311/TGF-β1/Snail1 signaling pathway	[226]
Compounds	4, 5, 7-14	*Sanghuang porus*	Extracted with ethyl acetate	Anti-microbial	Test microorganisms	in vitro	Bacillus subtilis(-), Micrococcus luteus(-), Staphylococcus aureus(-), Mucor hiemalis(-)	—	[78]

Lyophilized powder	—	*P. igniarius*	Extracted by decoction and with alchohol	Anti-bacterial	Turbidimetric method	in vitro	Absorbance at 600nm(-)	—	[98]
Silver nanoparticles with polysaccharides	CS-FSHPs-Ag2	Mycelia (*S. sanghuang*)	Extracted with hot water	Anti-bacterial	Staphylococcus aureus Escherichia coli	in vitro	Inhibition zone(+)	—	[111]
Mycelium extract	SvMEs	Mycelium (*S. vaninii*)	Extracted with 50% ethanol by 35 kHz ultrasonic assistance	Anti-Parkinson's Disease	Zebrafish PD model	in vivo	DA neurons(+), disorganisation(-), loss of vasculature(-), total distance travelled(+), swimming speed(+),	—	[32]
Polysaccharides	—	*P. igniarius*	Extracted with 95% ethanol	Improving immunity	SD mice	in vivo	Body weight(+), organ indices(+), white blood cell(+), hemoglobin(+), alkaline phosphatase(+), lactate dehydrogenase(+)	—	[200]
Proteoglycan	PL	*P. linteus*	Precipitated with ethanol	Regulating immunity	Female C57BL/6 mice	in vivo	Murine splenic lymphocytes(+), CD3+ cells(-), CD4+ cells(-), B cells(+), surface molecules on B cells (CD86 and CD80)(+), protein kinase C activity in B cells(+), tyrosine phosphorylation in B cells(+)	—	[56]
